# Au–Ag and Pt–Ag bimetallic nanoparticles@halloysite nanotubes: morphological modulation, improvement of thermal stability and catalytic performance†

**DOI:** 10.1039/c8ra00423d

**Published:** 2018-03-14

**Authors:** Siyu Li, Feng Tang, Huixin Wang, Junran Feng, Zhaoxia Jin

**Affiliations:** Department of Chemistry, Renmin University of China Beijing 100872 P. R. China jinzx@ruc.edu.cn

## Abstract

In this study, Au–Ag and Pt–Ag bimetallic nanocages were loaded on natural halloysite nanotubes (HNTs) *via* galvanic exchange based on Ag@HNT. By changing the ratio of Au to Ag or Pt to Ag in exchange processes, Au–Ag@HNT and Pt–Ag@HNT with different nanostructures were generated. Both Au–Ag@HNT and Pt–Ag@HNT systems showed significantly improved efficiency as peroxidase-like catalysts in the oxidation of *o*-phenylenediamine compared with monometallic Au@HNT and Pt@HNT, although inert Ag is dominant in the composition of both Au–Ag and Pt–Ag nanocages. On the other hand, loading on HNTs enhanced the thermal stability for every system, whether monometallic Ag nanoparticles, bimetallic Au–Ag or Pt–Ag nanocages. Ag@HNT sustained thermal treatment at 400 °C in nitrogen with improved catalytic performance, while Au–Ag@HNT and Pt–Ag@HNT maintained or even had slightly enhanced catalytic efficiency after thermal treatment at 200 °C in nitrogen. This study demonstrated that natural halloysite nanotubes are a good support for various metallic nanoparticles, improving their catalytic efficiency and thermal stability.

## Abstract 2

Bimetallic Au–Ag@HNT and Pt–Ag@HNT nanocages showed significantly improved efficiency in the oxidation of *o*-phenylenediamine as peroxidase-like catalyst compared with corresponding monometallic nanoparticles.

## Graphical abstract

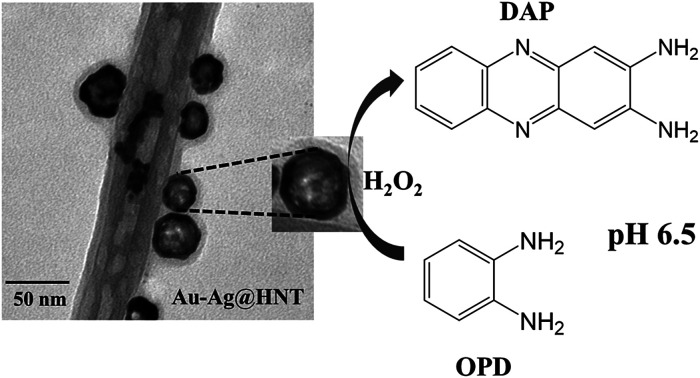

## Introduction

Metal nanoparticles (NPs) have great applications in the chemical industry and environmental science as heterogeneous catalysts. Compared with monometallic nanoparticles, bimetallic NPs have improved catalytic activity and durability in many reactions owing to the combined advantages of the bimetallic components at the atomic level.^1,2^ In particular, bimetallic nanocrystals display plenty of scope for modulation of their morphologies and compositions, providing a wide platform of specific catalysts.^1,3–6^ The Ag nanoparticle itself has demonstrated a variety of applications, based its catalytic activity, surface-enhanced Raman scattering and antibacterial action.^7,8^ Ag-based bimetallic nanocrystals Ag–M (M = Au, Pd or Pt) have shown significantly improved catalytic activity and extended the application landscape.^9–11^ For example, bimetallic Au–Ag NPs have demonstrated peroxidase-like activity, whereas monometallic Ag NPs are inert.^12^ However, coarsening of metal or bimetallic nanoparticles in catalytic processes under different reaction conditions induces a dramatic decrease of their catalytic efficiency. To avoid it, anchoring metal or bimetallic nanoparticles on supports with high surface area is a general solution. This introduces a new influential factor for catalyst efficiency, that is, the interaction between NP catalysts and their support.^13,14^ For example, the strain between nanoparticles and substrates may assist the catalytic process *via* reducing the energy barrier of reactions.^15^ Impressively, the interaction between metal nanoparticles and support surface may significantly enhance the thermal stability of metallic catalysts.^16^ Mesoporous supports with different Si/Al ratios have demonstrated distinct catalytic activities due to their different densities of active defects which play a key role in stabilizing and activating metal nanoparticles.^17^

Halloysite, a kind of natural clay (chemical composition: Al_2_Si_2_O_5_(OH)_4_·*n*H_2_O, 1 : 1 layer aluminosilicate) with tubular nanostructure, is promising as a catalyst support.^18–21^ Halloysite has many advantages, such as being environmentally benign, low cost and having large surface area. However, to anchor metal nanoparticles, specific surface modification of halloysite is generally required,^22–24^ and organosilane is often used as a surface modifier.^25–33^ But the introduction of small molecules as surface modifier on halloysite surface may disfavor the thermal stability of the metal@halloysite system owing to its thermal decomposition at high temperature, and its susceptibility in some organic solvents or varied pH conditions may also induce detachment of modifier molecules. These weaknesses have limited the application of metal@halloysite catalysts. Moreover, because of the elegant control of experimental conditions required in the fabrication of bimetallic nanoparticles,^34^ decorating bimetallic nanoparticles on halloysite nanotubes has seldom been achieved.

Herein, Ag@HNT was fabricated through a modified silver-mirror-reaction without pretreatment of HNT surface. Then porous bimetallic Au–Ag and Pt–Ag nanocages were successfully generated on halloysite *via* galvanic exchange of Ag@HNT. Structural and morphological characterizations showed that Au or Pt domains intertwine with Ag domains, and Ag is dominant in the composition of these Au–Ag and Pt–Ag nanocages on HNT. Bimetallic Au–Ag@HNT and Pt–Ag@HNT demonstrated significantly improved activity as peroxidase-like catalysts in the catalytic oxidation of *o*-phenylenediamine (OPD) in the presence of H_2_O_2_ compared with monometallic Au@HNT and Pt@HNT, where Ag@HNT is almost inert. In addition, the thermal stabilities of Ag@HNT, Au–Ag@HNT and Pt–Ag@HNT were carefully studied. The peroxidase-like catalytic ability of Au–Ag@HNT and Pt–Ag@HNT systems was maintained, or slightly improved, after thermal treatment at 200 °C for 20 min in N_2_ environment. Ag@HNT can resist thermal treatment at higher temperature (400 °C), to give even better catalytic efficiency in the reduction of 4-nitrophenol by NaBH_4_. The combination of Ag-based bimetallic nanostructures and natural halloysite will allow the development of catalysts with low cost and high efficiency, which are highly desirable for industrial applications.

## Experimental section

### Materials

Halloysite mineral (HNT) was purchased from Zhengzhou Jinyangguang Ceramics Co. Ltd. Pristine HNT was cleaned with fresh water, dried in a freeze-drier, and then ground into fine powder in a mortar before use. Analytical grade chloroauric acid (HAuCl_4_), chloroplatinic acid hexahydrate (H_2_PtCl_6_·6H_2_O), silver nitrate (AgNO_3_), 4-nitrophenol (4-NP) and ascorbic acid (AA) were purchased from Sinopharm Chemical Reagent Co. Ltd. Sodium borohydride (NaBH_4_, AR) was purchased from Tianjin Fuchen Chemical Reagent Technologies Co. Ltd. Glucose (AR) was purchased from Tianjin Fengchuan Chemical Reagent Technologies Co. Ltd. Ammonium hydroxide (NH_3_·H_2_O, 25%), ethanol (AR), ethylene glycol (EG, AR), hydrogen peroxide (H_2_O_2_, 30%) and sodium chloride (AR) were purchased from Beijing Chemical Works. Polyvinylpyrrolidone (PVP, MW 58 000, AR) was purchased from Alfa Aesar. *o*-Phenylenediamine (OPD, 99.9%) was purchased from Sigma. All chemicals were used as received without further purification.

### Preparation of Ag@HNT

Tollens' reagent (10 mM) was prepared as follows. First, NH_3_·H_2_O (2%) was added into AgNO_3_ solution (160 mM, 5 mL) to induce precipitation; then extra aqueous ammonia was added until the precipitate was completely dissolved; finally the solution was diluted to 80 mL with deionized water. Cleaned halloysite nanotubes (0.16 g), glucose (6.40 g) and PVP (1.60 g) were dispersed in EG (80 mL). After mixing, 10 mM freshly prepared Tollens' reagent (80 mL) was dropped into the above solution. The mixture was vigorously stirred in a 40 °C water bath for 30 min. The reaction time was shortened to 10 min when the preparation temperature was changed to 60 °C or 80 °C. Then the suspension was centrifuged (8000 rpm, 10 min) and washed three times with deionized water and ethanol, respectively. The as-prepared Ag@HNT was dried at 65 °C in an oven overnight.

The preparation methods of Au NPs, Pt NPs, Au@HNT, and Pt@HNT samples are shown in the ESI.†

### Preparation of Au–Ag@HNT and Pt–Ag@HNT

The galvanic exchange method was followed for the synthesis of bimetallic nanoparticles based on Ag@HNT. Ag@HNT (5.2 mg) was dispersed in PVP solution (1 mg mL^−1^, 100 mL) in a 50 °C water bath. Then, HAuCl_4_ (10 mM) aqueous solution (200 μL) was slowly dropped into the above solution under vigorous magnetic stirring, such that the added Au : Ag atomic ratio was 0.1. The color of the solution changed from brown to brownish red within several seconds, indicating the formation of Au–Ag@HNT *via* galvanic exchange. The composition of Au–Ag@HNT was tuned by adjusting the ratio of Ag@HNT to HAuCl_4_ (Table S1†). The reaction solution was stirred for 5 min and centrifuged at 10 000 rpm for 10 min.

For the synthesis of Pt–Ag@HNT, a similar procedure was followed except H_2_PtCl_6_·6H_2_O (10 mM, 200 μL) was used as precursor, instead of HAuCl_4_. The color of the solution changed from brown to grayish black within several minutes, showing the formation of Pt–Ag@HNT. The composition of Pt–Ag@HNT was adjusted by changing the ratio of Ag@HNT to H_2_PtCl_6_ (Table S1†). The reaction mixture was stirred for 10 min and centrifuged at 12 000 rpm for 10 min.

Finally, both Au–Ag@HNT and Pt–Ag@HNT were washed twice with ethanol, saturated sodium chloride solution and then deionized water. Then the samples were dried at 65 °C in an oven overnight.

### Thermal treatment

The dried powders of Ag@HNT, Au–Ag@HNT and Pt–Ag@HNT were heated under nitrogen flow (40 cm^3^ min^−1^) from 25 °C to 200 °C (or 400 °C) at a heating rate of 10 °C min^−1^, and kept at 200 °C (or 400 °C) for 20 min.

### Characterizations of metallic nanoparticles and their composites with HNTs

Nanoparticles and M@HNTs samples were dispersed in water to form suspensions by sonication. Then a drop of each suspension was placed onto a copper grid for transmission electron microscopy (TEM) characterization (Hitachi TEM, H-7650B) at an accelerating voltage of 100 kV. Scanning electron microscopy (SEM) characterization was conducted by using an SU8010 scanning electron microscope. High-resolution transmission electron microscopy characterization (HRTEM) was performed by using a Tecnai G2 F20 (FEI) microscope at an accelerating voltage of 200 kV. Energy-dispersive X-ray analysis (EDX) element mapping was conducted in high-angle annular dark field (HAADF) mode with the same microscope.

UV-vis absorption spectra of metallic nanoparticles and their composites with halloysites were measured by using a Varian Cary 50 spectrometer. Wide-angle X-ray diffraction was performed with an XRD-7000 diffractometer (Shimadzu) in the reflection mode using a Cu target to produce incident X-rays (*λ* = 0.15418 nm). The scan speed was 2° min^−1^, and the scan scope was from 10° to 80°. The Ag, Au and Pt contents of these samples were determined by using inductively coupled plasma optical emission spectroscopy (ICP-OES) (Agilent ICP/700). Before characterization, samples (1 mg) were dissolved in an aqua regia solution (1 : 3 v/v HNO_3_ : HCl, 10 mL) for 3 days. Then, 0.5 mL of the aqua regia solution was diluted to 10 mL with deionized water. The final concentration of the test liquid was 5.0 mg L^−1^.

### Catalytic performance

#### Catalytic reduction of 4-NP by NaBH_4_

The catalytic efficiency of M@HNT in the reduction of 4-NP in the presence of NaBH_4_ was investigated by using UV-vis spectrometry. It was observed that a red shift of the absorption peak due to 4-NP from 317 to 400 nm occurred immediately after the addition of NaBH_4_. This was because of the formation of 4-nitrophenolate ion in alkaline conditions caused by NaBH_4_. After adding M@HNT to this solution, the intensity of the peak (400 nm) of the nitro compound was successively decreased, and a new peak at 295 nm appeared due to the formation of 4-aminophenol (4-AP). The catalytic efficiency was quantitatively calculated based on the successively decreased intensity of the peak at 400 nm with time.^35^ In a typical reaction, NaBH_4_ (1.70 mg) was added to the 4-NP aqueous solution (0.1 mM, 15 mL) in a round flask with vigorous magnetic stirring, then Ag@HNT (7.5 mg) or bimetallic NPs@HNT (4.5 mg) was added. The mixture was sampled after every 2 min and the absorption spectra of the samples were recorded after centrifugation (14 000 rpm, 2 min).

#### Peroxidase-like catalytic oxidation of OPD

Ag-based bimetallic NPs can quickly catalyze the oxidation of OPD, a typical horseradish peroxidase (HRP) substrate, in the presence of H_2_O_2_. The oxidation rates were evaluated by monitoring the absorbance at 418 nm originating from DAP (2,3-diaminophenazine), the oxidized product of OPD. The catalytic oxidation of OPD by bimetallic NPs@HNT in the presence of H_2_O_2_ was performed as follows: 10 μL of 0.1 M OPD aqueous solution (freshly prepared) and bimetallic NPs@HNT (0.2 mg) were added to 3 mL of H_2_O at 40 °C. Then, H_2_O_2_ (30%, 100 μL) was added to the mixture. The pH of the mixture was 7. The oxidation reaction progress was monitored by recording the UV-vis absorption spectra of the solution at 2 min intervals.

## Results and discussion

### The fabrication and catalytic performance of Ag@HNT, Au–Ag@HNT and Pt–Ag@HNT

**Scheme 1:**
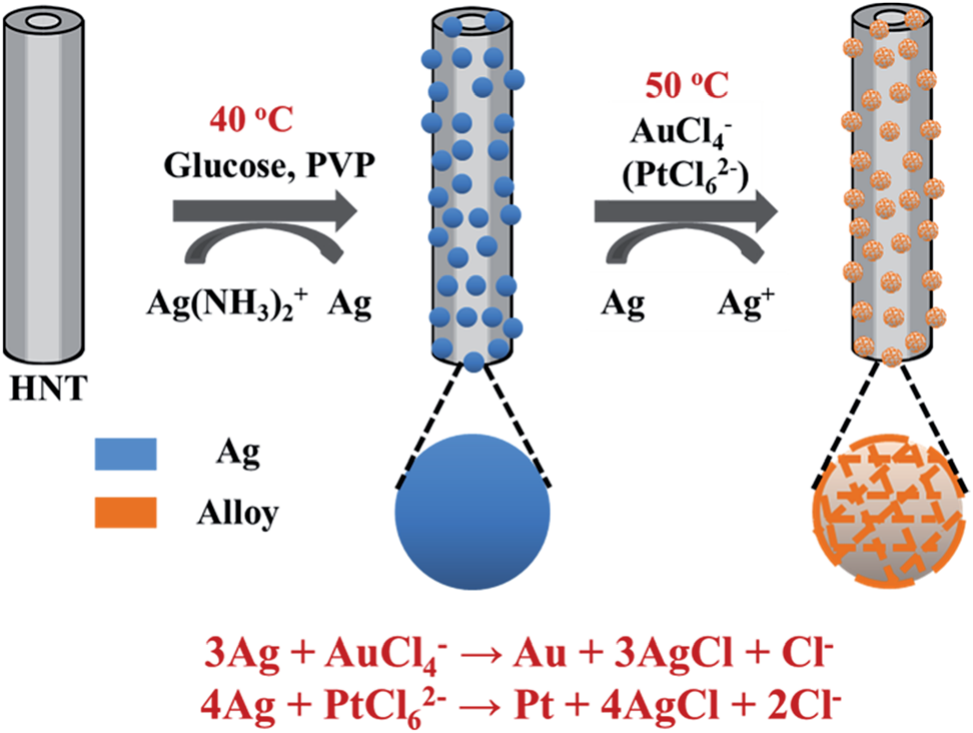
Schematic illustration of the fabrication of Ag@HNT, Au–Ag@HNT, and Pt–Ag@HNT.

**Fig. 1:**
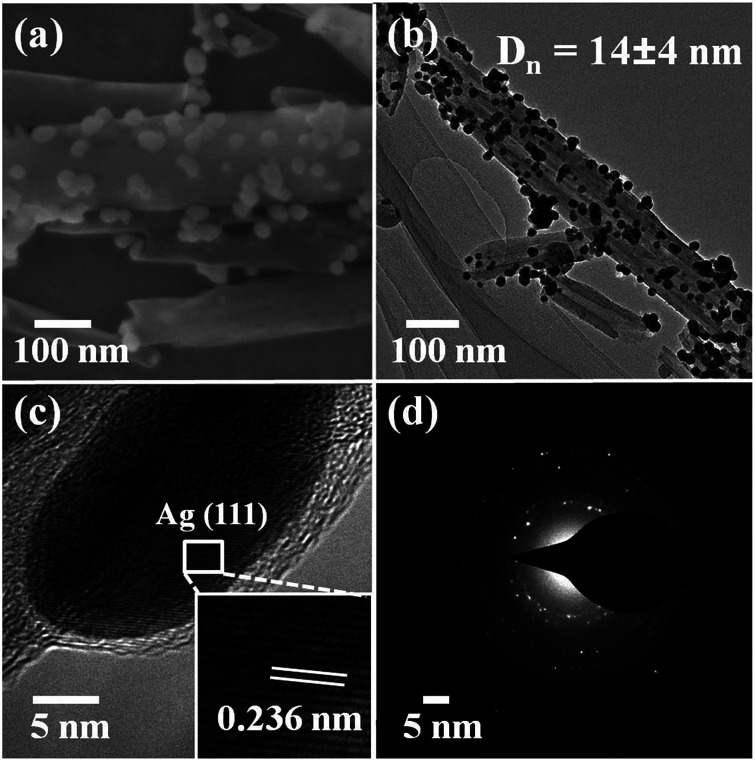
(a) SEM image of Ag@HNT with densely decorated Ag NPs. (b) TEM image of Ag@HNT, demonstrating Ag nanoparticles with well-controlled diameter. (c) HRTEM image of Ag@HNT, showing well-crystallized Ag with cover layer. (d) SAED pattern of Ag@HNT.

To decorate Ag nanoparticles on raw halloysite, the silver-mirror process was modified (Scheme 1). For improving the stability of Ag@HNT, we added PVP to the reaction medium. Glucose was the reducing agent for Ag ions. In addition, we noticed that different temperatures (40 °C, 60 °C or 80 °C) showed significant influence on the size distribution of Ag nanoparticles on HNTs. Higher temperature accelerated the growth of Ag NPs, resulting in large size and wide size-distribution of Ag NPs (Fig. S1†). The modified silver-mirror process at 40 °C showed great success in loading Ag nanoparticles on untreated halloysite nanotubes (Fig. 1). The mean diameter of fabricated Ag NPs was about 14 nm (Fig. 1b). HRTEM and selected area electron diffraction (SAED) showed that the obtained Ag nanoparticles on HNT were well crystallized (Fig. 1c and d).

**Fig. 2:**
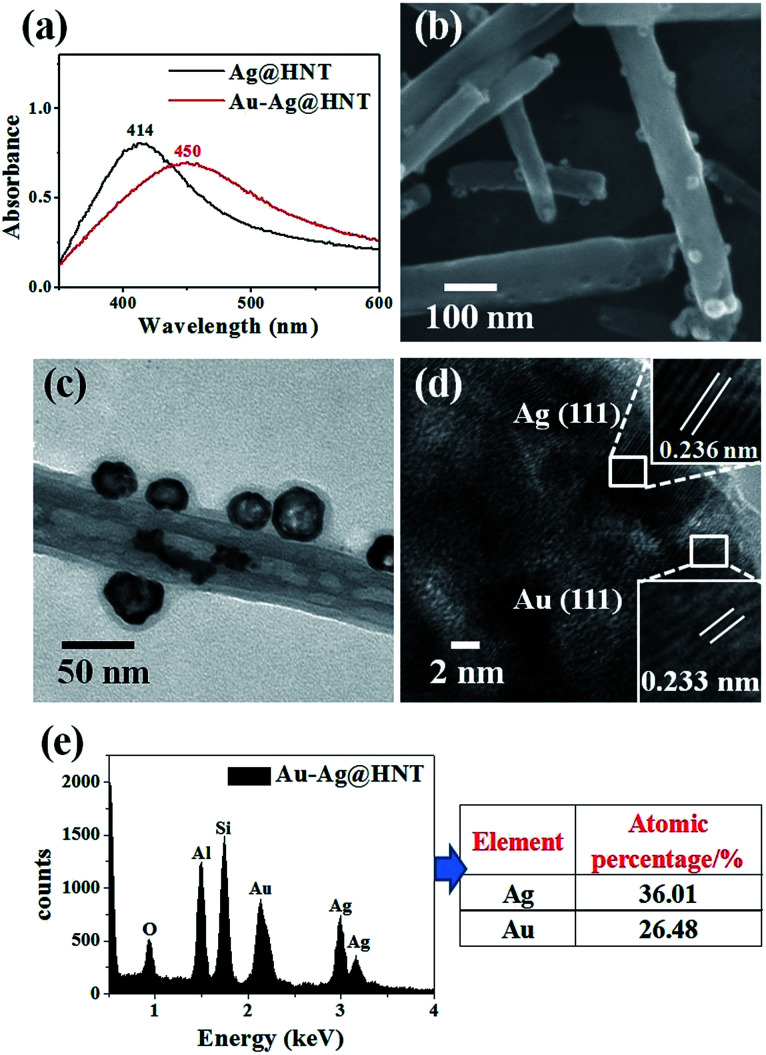
(a) UV-vis spectra of Ag@HNT and Au–Ag@HNT. (b) SEM image of Au–Ag@HNT. (c) TEM image of Au–Ag@HNT. (d) HRTEM image of Au–Ag@HNT, identifying the co-existence of Au and Ag. (e) EDX spectrum of Au–Ag@HNT and the corresponding atomic percentages of Au and Ag. The added atomic ratio of Au : Ag was 0.1.

We noticed that the presence of halloysite significantly improved the size control of Ag nanoparticles. Under the same experimental conditions only without halloysite, the size of Ag nanoparticles was about 40–50 nm in diameter and they aggregated heavily (Fig. S2†). Using these Ag@HNT samples (generated at 40 °C), we fabricated bimetallic Au–Ag@HNT and Pt–Ag@HNT. Scheme 1 presents the fabrication procedure of Ag@HNT and bimetallic Au–Ag@HNT and Pt–Ag@HNT nanocomposites. Galvanic exchange is commonly used in the generation of bimetallic nanoparticles or even trimetallic nanostructures, in which the morphology of bimetallic nanoparticles varies with experimental conditions.^36–39^ HAuCl_4_ or H_2_PtCl_6_ was added separately to Ag@HNT suspension to generate galvanic exchange. The shift or disappearance of surface plasmon resonance (SPR) of the original Ag nanoparticles indicated the formation of Au–Ag bimetallic nanoparticles (Fig. 2a). The etching of HAuCl_4_ induced a slight decrease of the loading amount of silver nanoparticles on HNT and significant hollowing of solid silver nanoparticles (Fig. 2b, c). Silver nanoparticles on halloysites became hollow Au–Ag nanocages. The content of gold and silver in the Au–Ag@HNT was checked by EDX (Fig. 2e). Silver remained dominant in the composition of bimetallic Au–Ag nanocages. Increasing the added atomic ratio of Au to Ag in this galvanic exchange intensified the etching of solid Ag by HAuCl_4_. The morphology of Au–Ag nanostructures on halloysite nanotubes can be modulated from nanocages to network-like nanostructures by changing the atomic ratio of Au to Ag from 0.1 to 1 (Fig. 2, S3a, S3b†). The corresponding SPR absorption of bimetallic nanoparticles also changed with the atomic ratio of Au : Ag, from 450 nm (0.1, corresponding to nanocages) to 527 nm (1, corresponding to network-like nanostructure). A further increase of the ratio of Au to Ag induced broken nanostructure (Fig. S3c†). ICP-OES characterizations showed the weight percentage of Au : Ag in the bimetallic nanostructures obtained with added atomic ratios from 0.1 to 1 (Fig. S4a†).

**Fig. 3:**
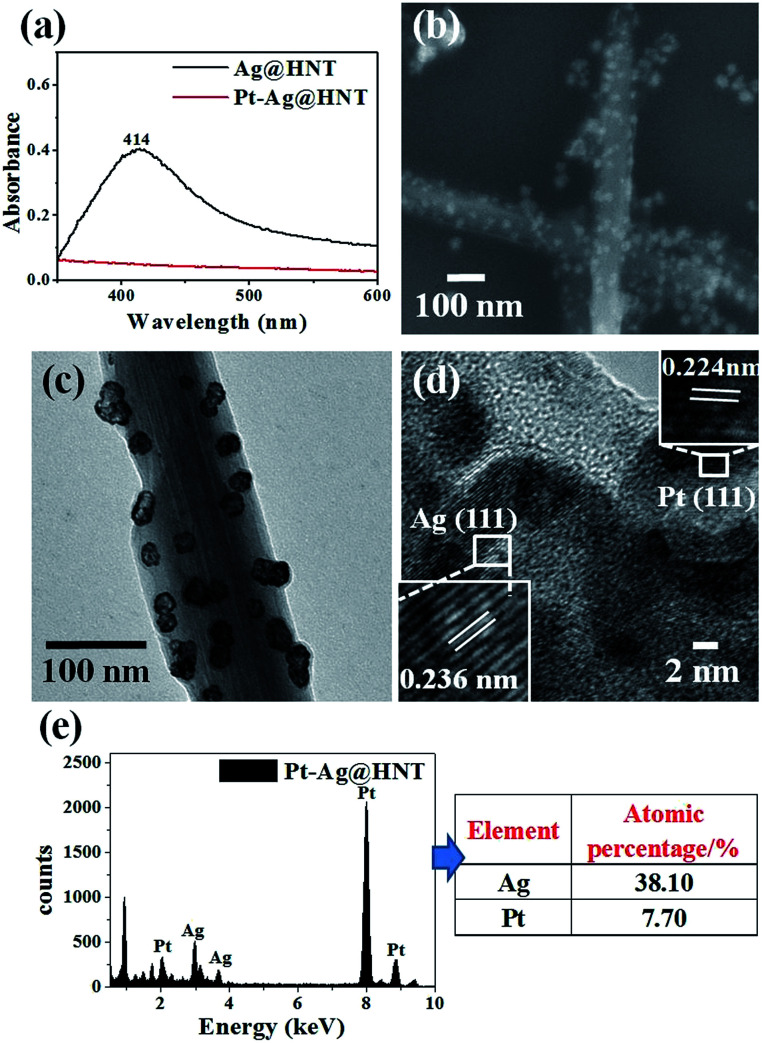
(a) UV-vis spectra of Ag@HNT and Pt–Ag@HNT. (b) SEM image of Pt–Ag@HNT. (c) TEM image of Pt–Ag@HNT. (d) HRTEM image of Pt–Ag@HNT, identifying the co-existence of Au and Ag. (e) EDX spectrum of Pt–Ag@HNT and the corresponding atomic percentages of Pt and Ag. The added atomic ratio of Pt : Ag was 0.1.

The morphological and structural characterizations of Pt–Ag@HNT are shown in Fig. 3. In the case of the fabrication of the Pt–Ag system, Pt–Ag nanocages were generated with added atomic ratio of Pt : Ag ∼0.1 (Fig. 3c) in which the weight percentage of Ag measured by ICP-OES was nearly double the percentage of Pt (Fig. S4b†). HRTEM characterization presented crystallized Pt domains intertwined with Ag domains (Fig. 3d). Combining the morphological and composition information, we observed that when the weight percentage of Pt is over that of Ag in bimetallic nanostructures, the complete Pt–Ag nanocages lose their cage-like morphology (Fig. S5†). XRD patterns of Au–Ag@HNT and Pt–Ag@HNT also supported the existence of bimetallic nanoparticles (Fig. S6†).

**Fig. 4:**
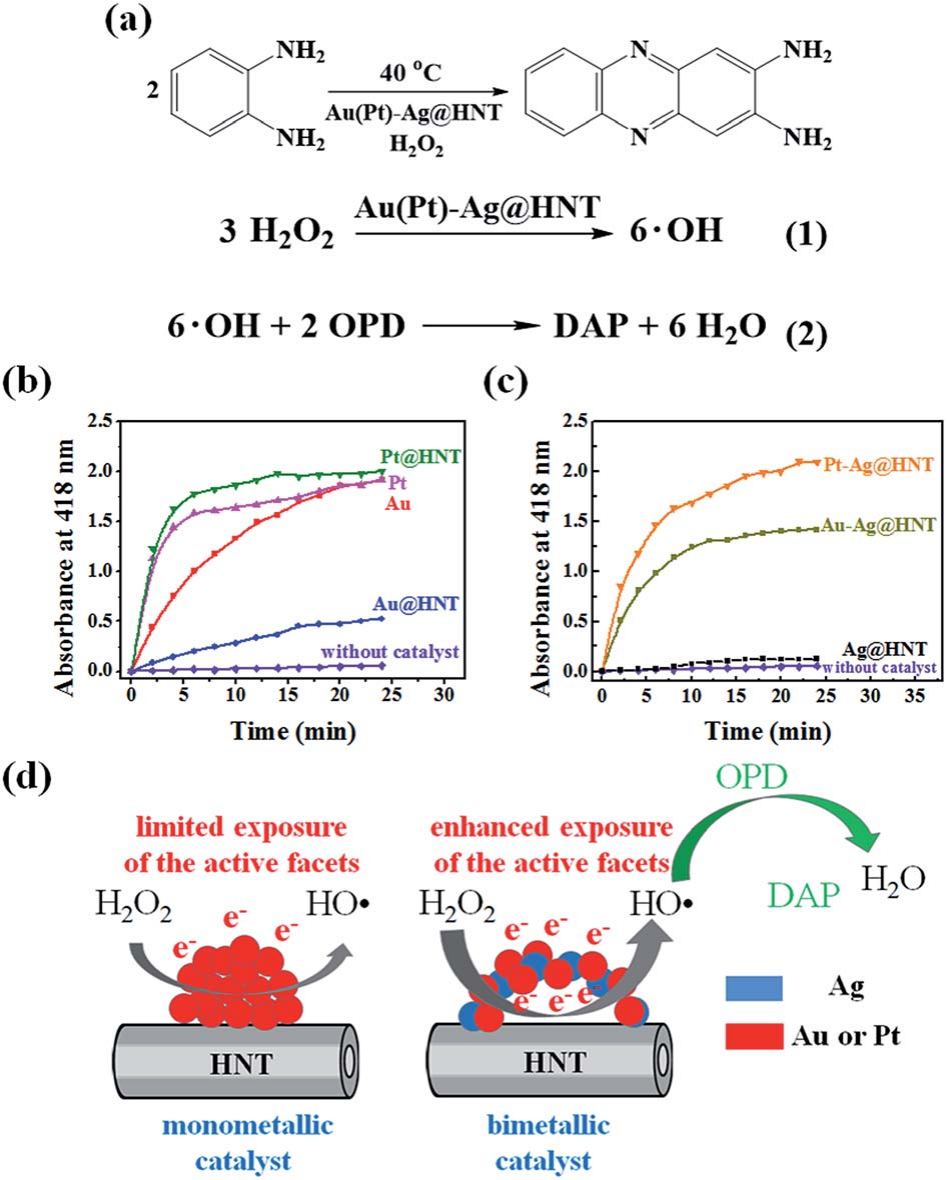
(a) The proposed reaction mechanism of the transformation of OPD to DAP. (b) Peroxidase-like catalytic performance of Au, Pt, Au@HNT, and Pt@HNT. The purple line shows the data from the control experiment. (c) Peroxidase-like catalytic performance of Ag@HNT, Au–Ag@HNT and Pt–Ag@HNT. Absorbance at 418 nm was monitored as a function of time after adding catalysts (0.2 mg). Reaction conditions: H_2_O_2_ (0.3 M), OPD (0.3 mM), and catalyst (0.2 mg) at 40 °C. Au(Pt)–Ag@HNT was prepared with the added atomic ratio of Au(Pt) : Ag of 0.1. (d) Schematic diagram of monometallic catalyst (with limited exposure of the active facets of metallic nanoparticles) and bimetallic catalyst (with enhanced exposure of the active facets of metallic nanoparticles) in the peroxidase-like catalytic oxidation of OPD.

The formation of bimetallic nanocages on halloysite nanotubes significantly extended and improved the catalytic ability of Ag@HNT. Bimetallic Au–Ag and Pt–Ag demonstrated peroxidase-like catalytic activity in the oxidation of OPD in the presence of H_2_O_2_, in which Ag itself was almost inert.^40^ H_2_O_2_ can oxidize OPD to form 2,3-diaminophenazine (DAP, yellow color), showing a new absorbance peak at 418 nm (Fig. 4a).^41^ Monitoring the absorbance change at 418 nm is the common way to investigate this reaction. We propose the reaction process from OPD to DAP in the presence of H_2_O_2_ on the surfaces of mono- or bi-metallic NPs as shown in eqn (1) and (2) in Fig. 4a based on the literature.^42^ Monometallic Au and Pt nanoparticles, loaded on HNT or not, showed a catalytic effect in the oxidation of OPD. Pt@HNT showed higher catalytic efficiency than Au@HNT, but the performances of their unloaded nanoparticles were nearly the same (Fig. 4b). Fig. 4c presents the catalytic performance of Au–Ag@HNT and Pt–Ag@HNT compared with Ag@HNT. Although Ag@HNT had nearly no catalytic effect in this reaction, both Au–Ag@HNT and Pt–Ag@HNT demonstrated high catalytic efficiency. Using the reported molar absorption coefficient of the neutral form of DAP, *ε*_418_ = 16 700 M^−1^ cm^−1^,^43^ it can be estimated that 74.3% of the OPD was transformed into DAP in the first 20 min in the presence of Pt–Ag@HNT, which showed much higher catalytic efficiency than Au–Ag@HNT. The surface properties of Pt–Ag@HNT would influence the absorption of H_2_O_2_ and the particle-mediated electron transfer, promoting H_2_O_2_ to break up into HO˙ radicals.^42^ Because halloysite and Ag have nearly no catalytic effect, for comparing the reaction rate in different catalyst systems, the reaction rate constant (*k*) in each system was divided by the molar value of the active elements (calculated on the basis of ICP-OES results), which are Au in both Au@HNT and Au–Ag@HNT, and Pt in both Pt@HNT and Pt–Ag@HNT, to generate k* shown in Table 1.

**Table 1:** The DAP formation rate constant k* (normalized based on the molar content of active elements) in different catalytic systems

Sample		k* (mol-DAP per min per mol-M)
Au NPs		0.03
Au@HNT		0.04
Pt NPs		0.03
Pt@HNT		0.16
Au–Ag@HNT	Au : Ag = 0.1	0.39
	Au : Ag = 0.5	0.23
	Au : Ag = 1	0.12
Pt–Ag@HNT	Pt : Ag = 0.1	0.99
	Pt : Ag = 0.5	0.95
	Pt : Ag = 1	0.48

The improvement in catalytic performance produced by loading on a support can be assigned to the influence of morphology and electronic structure of metallic nanoparticles being changed by the support's properties.^44,45^ In the preparation of Ag NPs and Ag@HNT, we found that HNT supports were favorable to the size control of loaded Ag NPs. On the other hand, it is reported that Au nanoparticles are easily faceted while anchored on the support surface compared with in solution.^46^ Loading on halloysite improved the catalytic performance of monometallic Au or Pt nanoparticles (Table 1), and forming bimetallic Au–Ag@HNT and Pt–Ag@HNT further enhanced their catalytic efficiency. The DAP formation rate in the case of Au–Ag@HNT was over 13 times that in the Au@HNT system, and k* for Pt–Ag@HNT was 6 times that in the Pt@HNT case (Table 1). This indicated the active components were much more efficient while they were separated by the Ag spacer. Separated Au or Pt may favor the most exposure of the active facets (Fig. 4d). The Au–Ag@HNT and Pt–Ag@HNT catalysts were recycled 4 times, and the corresponding DAP formation rate constants are shown in Fig. S7.† The k* value decreased slightly after each cycle, which might partially be attributed to the sample loss in the recycling process.

The catalytic efficiencies of Au–Ag@HNT and Pt–Ag@HNT generated with different experimental parameters were also compared (Table 1, Fig. S8†). An increase of Au or Pt amount induced a decrease of catalytic efficiency, which may be because of the damage to perfect bimetallic nanocages at higher atomic ratios of Au(Pt) : Ag (Fig. S3, S5†), and the decrease of Ag amount (Fig. S4†). If the Ag spacer is lost, the active domains of Au or Pt may aggregate together, leading to a reduction in catalytic efficiency. Bimetallic nanocages of Au–Ag@HNT or Pt–Ag@HNT, in which there are enough Ag spacers between Au or Pt domains, showed better performance than those bimetallic nanostructures with less Ag. In addition, the catalytic performances of Ag@HNT, Au–Ag@HNT and Pt–Ag@HNT were compared in the reduction of 4-NP in the presence of NaBH_4_, presenting a universal improvement for bimetallic systems (Fig. S9†).

### The influence of thermal treatment of metal NPs-HNT composites

**Fig. 5:**
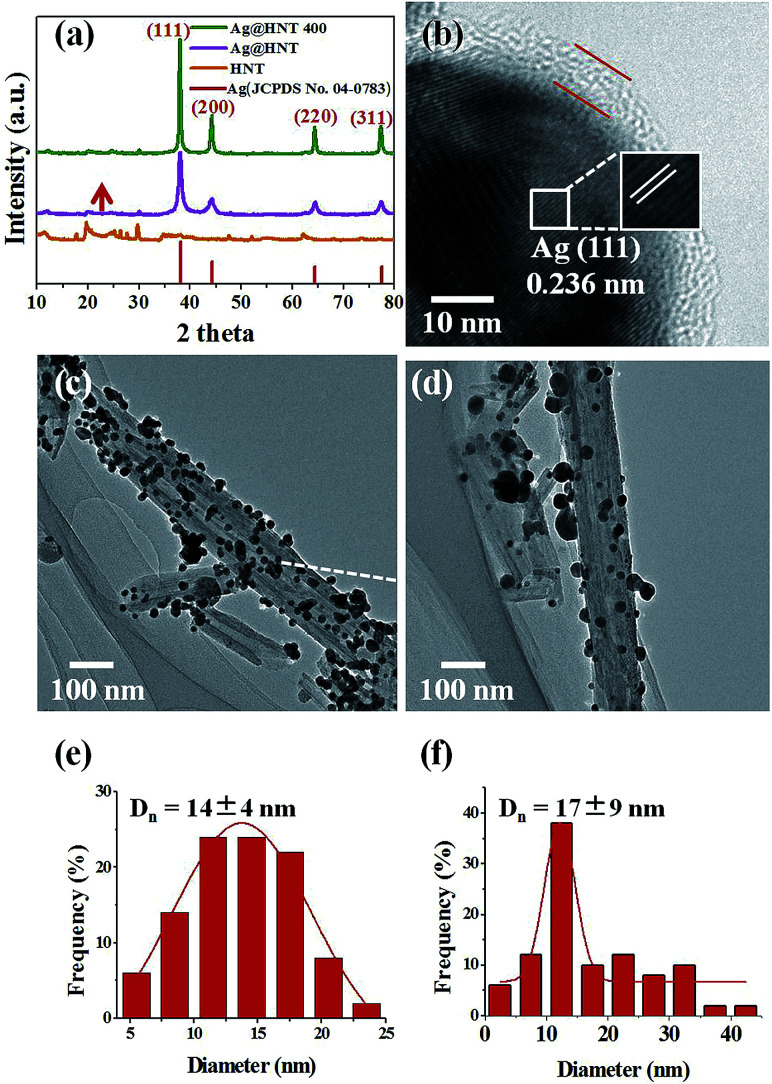
(a) Powder X-ray diffraction (XRD) patterns of HNT and of Ag@HNT before and after heating to 400 °C under nitrogen. (b) HRTEM image of Ag@HNT heated to 400 °C under nitrogen. TEM images of Ag@HNT before (c) and after (d) heating to 400 °C under nitrogen. Size distributions of Ag nanoparticles before (e) and after (f) heating to 400 °C under nitrogen.

The thermal stabilities of Ag@HNT, Au–Ag@HNT and Pt–Ag@HNT were explored. Fig. 5 presents morphological and structural information concerning Ag@HNT nanocomposites after thermal treatment at 400 °C in N_2_ atmosphere for 20 min.

**Fig. 6:**
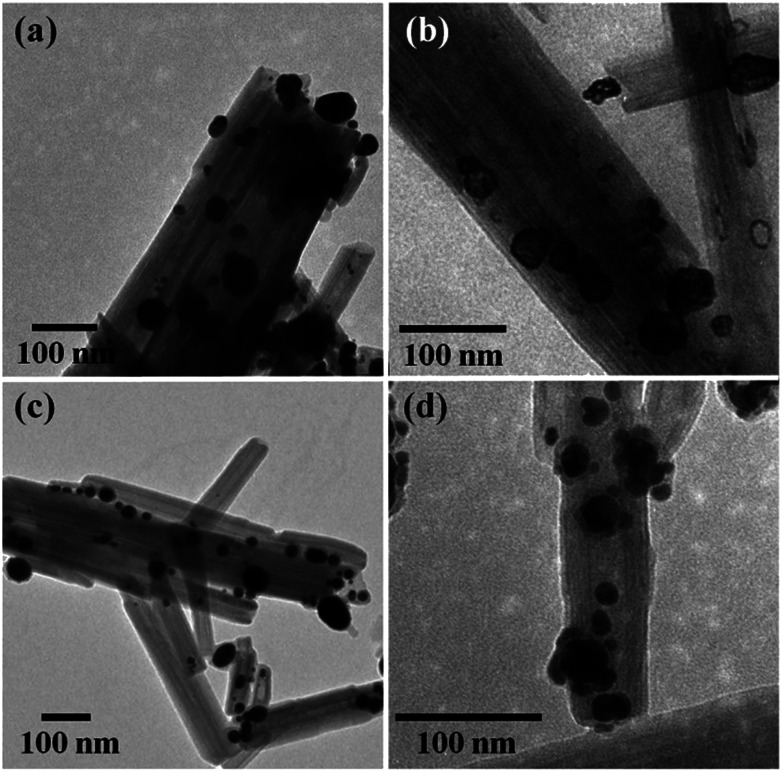
TEM images of Au–Ag@HNT (a) and Pt–Ag@HNT (b) after heating to 200 °C under nitrogen, and Au–Ag@HNT (c) and Pt–Ag@HNT (d) after heating to 400 °C under nitrogen. Au(Pt)–Ag@HNT was prepared with the added atomic ratio of Au(Pt) : Ag of 0.1.

Thermal treatment induced a change of size distribution of Ag on halloysite. The number of larger particles increased, which was supported by the sharper peak of the (111) Ag crystal plane in the XRD pattern of heated Ag@HNT (Fig. 5a). But the loading amount of silver on HNT showed no significant change (Table 2). HRTEM characterization revealed that carbon was derived from pyrolysis of polymeric stabilizer covering Ag NPs and halloysites, and it may behave as a buffer to stop the merging of Ag NPs on halloysite (Fig. 5b). The composition of Ag@HNT, Au–Ag@HNT and Pt–Ag@HNT demonstrated no distinct change before and after heating to 200 °C (400 °C) based on the measurement by ICP-OES (Table 2). In the case of bimetallic Au–Ag@HNT, Au–Ag nanocages became solid bimetallic nanoparticles, whereas Pt–Ag@HNT showed no distinct morphological change after thermal treatment at 200 °C. Increasing the temperature of the thermal treatment (400 °C) showed significant influence on loading densities for both Au–Ag and Pt–Ag systems (Fig. 6). The polymer layer covering Ag nanoparticles protected against the merging of Ag NPs during heating to 400 °C, but this protection was lost in the case of bimetallic Au–Ag or Pt–Ag nanostructures. The severely decreased loading density of bimetallic NPs on HNT compared with that of Ag@HNT may be caused by damage to the polymer cover during the fabrication of Au–Ag@HNT or Pt–Ag@HNT. On the other hand, bimetallic nanoparticles experienced reconstruction during thermal treatment at 400 °C owing to the different surface energies of the two metals.^47^ For Au–Ag@HNT, the bimetallic nanoparticles changed from intertwined bimetallic nanocages to Ag-shell–Au-core nanostructures because of the lower surface energy of Ag, which could also induce damage of the covering layer of the original Ag NPs.

**Table 2:** The content of Ag, Au and Pt in Ag@HNT, Au–Ag@HNT and Pt–Ag@HNT before and after heating to 200 °C (or 400 °C) under nitrogen, measured by ICP-OES^a^

Temperature (°C)	Ag@HNT (wt%)	Au–Ag@HNT (wt%)	Pt–Ag@HNT (wt%)
RT^b^	Ag: 39.3 ± 0.9	Au: 5.7 ± 0.8	Pt: 4.9 ± 0.7
		Ag: 19.9 ± 0.6	Ag: 17.5 ± 0.7
200	Ag: 39.1 ± 1.0	Au: 6.2 ± 1.0	Pt: 3.6 ± 1.3
		Ag: 19.6 ± 0.6	Ag: 14.4 ± 0.1
400	Ag: 38.8 ± 0.8	Au: 7.9 ± 1.1	Pt: 4.0 ± 0.7
		Ag: 18.8 ± 0.6	Ag: 18.9 ± 0.2

a Au(Pt)–Ag@HNT was prepared with the added atomic ratio of Au(Pt) : Ag of 0.1.

b RT, Room temperature.

**Fig. 7:**
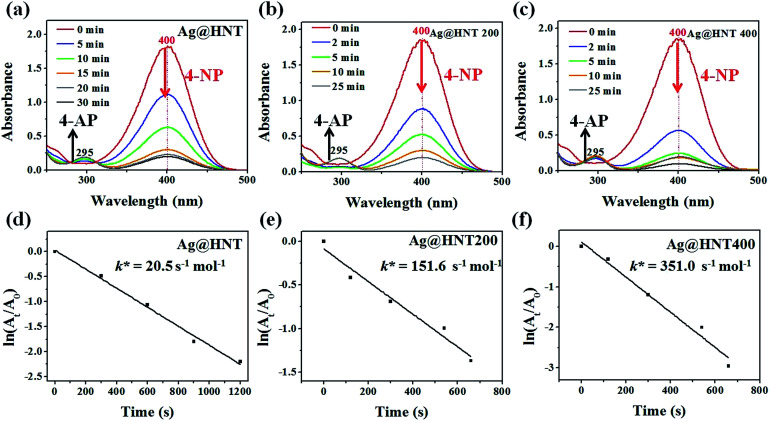
Time-dependent UV-vis absorption spectra for the catalytic reduction of 4-NP by NaBH_4_ in different catalytic systems. (a) In the presence of Ag@HNT (the amount of catalyst was 7.5 mg). (b) In the case of Ag@HNT 200. (c) In the case of Ag@HNT 400. The amounts of Ag@HNT 200 and Ag@HNT 400 catalyst were 4.5 mg. (d, e, f) The corresponding plots of ln(*A*_*t*_/*A*_0_) against time for the reduction of 4-NP in different catalytic systems.

Surprisingly, Ag@HNT after thermal treatment at 400 °C showed enhanced catalytic performance (Fig. 7). The conversion of 4-NP to 4-AP was faster than that with the original Ag@HNT even though the added amount of treated Ag@HNT was smaller. After thermal treatment (400 °C), the 4-NP reduction rate constant k* (normalized as *k* mol^−1^) was approximately 17 times the original one (Table S2,†Fig. 7). All heated Ag@HNT samples, no matter whether generated at 40, 60 or 80 °C, showed improved catalytic efficiency compared with untreated systems (Table S2†). We suppose that the partly carbonized PVP on Ag nanoparticles after thermal treatment may contribute to this improvement through accelerating the charge transfer.

**Fig. 8:**
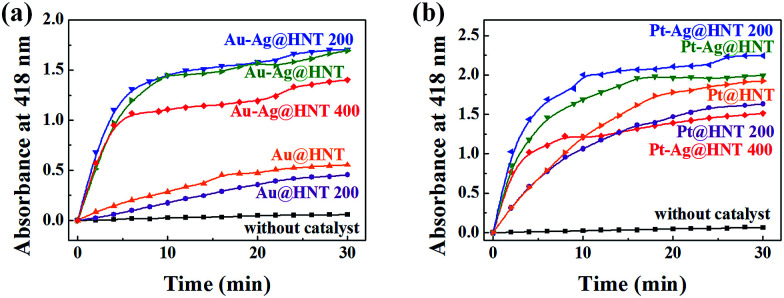
(a) Peroxidase-like catalytic performance of Au@HNT and Au–Ag@HNT before and after heating to 200 °C (or 400 °C) under nitrogen. (b) Peroxidase-like catalytic performance of Pt@HNT and Pt–Ag@HNT before and after heating to 200 °C (or 400 °C) under nitrogen. The black line shows the control experiment. Absorbance at 418 nm was monitored as a function of time after adding catalysts (0.2 mg). Reaction conditions: H_2_O_2_ (0.3 M), OPD (0.3 mM), and catalyst (0.2 mg) at 40 °C. Au(Pt)–Ag@HNT was prepared with the added atomic ratio of Au(Pt) : Ag of 0.1.

Bimetallic nanoparticles (Ag–Au and Au–Cu) with porous nanostructure and larger cavities have shown better catalytic performance than those with solid morphology in the literature reports.^48–50^ Based on the morphological characterization of treated Au–Ag@HNT, we noticed that porous Au–Ag@HNT became solid Au–Ag@HNT after heating at 200 °C. On comparing the peroxidase-like catalytic efficiency, we found that solid Au–Ag@HNT (200 °C) maintained the same catalytic efficiency as porous Au–Ag@HNT, although solid Au–Ag@HNT (400 °C) showed lower catalytic efficiency (Fig. 8). The influence of thermal treatment of Pt–Ag@HNT catalyst presented a similar result, which was that catalyst performance was slightly enhanced after heating to 200 °C whereas it declined after higher temperature treatment (400 °C). The catalytic efficiency of corresponding monometallic Au@HNT and Pt@HNT systems before and after thermal treatment at 200 °C was also compared (Fig. 8). Both Au@HNT 200 and Pt@HNT 200 presented decreased catalytic efficiency, which clearly highlighted the positive effect of bimetallic systems. Since the catalytic performance of metal nanoparticles directly relates to their specific crystalline facets, improved crystallization of bimetallic nanostructure after 200 °C treatment may contribute to their improved catalyst performance; but the loss of Ag spacers due to melting during thermal treatment at 400 °C offsets this benefit, resulting in lower catalytic efficiency.

## Conclusions

In this study, bimetallic Au–Ag or Pt–Ag nanocages loaded on halloysite nanotubes were generated on the basis of Ag@HNT, which was fabricated *via* a modified silver-mirror-reaction without any pretreatment of halloysite. The morphology of Au–Ag or Pt–Ag nanostructures on HNTs was modulated by changing the ratio of HAuCl_4_ (or H_2_PtCl_6_) to Ag in galvanic exchanges. The obtained bimetallic Au–Ag@HNT and Pt–Ag@HNT systems presented an improved and extended catalytic effect compared with Ag@HNT. The thermal stability of Ag@HNT, Au–Ag@HNT and Pt–Ag@HNT was explored as regards morphology, composition and catalytic performance. The Ag@HNT system had improved catalytic performance even after thermal treatment at 400 °C for 20 min as no obvious agglomeration and exfoliation of Ag nanoparticles on HNT were found, which may be attributed to the buffering effect of polymer covering on Ag nanoparticles during thermal treatment. However, the galvanic exchange process in the fabrication of bimetallic Au–Ag and Pt–Ag on halloysite may damage this polymer cover, resulting in a significant decrease of loading density of bimetallic nanostructures after thermal treatment at 400 °C, and lowering catalytic performance. But moderate thermal treatment at 200 °C did not decrease catalytic efficiency of bimetallic Au–Ag@HNT and Pt–Ag@HNT.

## Conflicts of interest

There are no conflicts to declare.

## Supplementary Material

RA-008-C8RA00423D-s001
