# Supplementary material for: Au–Ag and Pt–Ag bimetallic nanoparticles@halloysite nanotubes: morphological modulation, improvement of thermal stability and catalytic performance
Source: RSC Adv. 2018 Mar 14;8(19):10237–45. doi: 10.1039/c8ra00423d (PMC9078928; doi:10.1039/c8ra00423d)
Supplement: RA-008-C8RA00423D-s001 [file RA-008-C8RA00423D-s001.pdf]

Electronic Supplementary Material (ESI) for RSC Advances.
This journal is © The Royal Society of Chemistry 2018

Supporting Information

Au-Ag and Pt-Ag Bimetallic Nanoparticles @Halloysite Nanotubes: the Morphological Modulation, Improvement of Thermal Stability and Catalytic Performance

Siyu Li, Feng Tang, Huixin Wang, Junran Feng and Zhaoxia Jin*

Department of Chemistry, Renmin University of China, 100872, P. R. China

Contents

Preparation method

1. Preparation of Au NPs and Au@HNT..4

2. Preparation of Pt NPs and Pt@HNT 4

Tables

Table S1. The experimental parameters in the synthesis of bimetallic nanoparticles@HNTs. 5

Table S2. The 4-NP reduction rate constant *k** in the presence of Ag@HNT prepared at 40 ^{o}C, 60 ^{o}C, and 80 ^{o}C before and after heating at 400 ^{o}C under nitrogen..............6

Table S3. The comparison of DAP formation rate constant k* (normalized based on the mole of active elements) in our catalytic systems and that reported in literatures. 6

Figures

Fig. S1. (a, b, c) TEM images of Ag@HNTs prepared at 60 ^{o}C (a), 80 ^{o}C (b), and 100 ^{o}C (c). (d, e, f). Size distribution of Ag nanoparticles prepared at 60 ^{o}C (d), 80 ^{o}C (e), and 100 ^{o}C (f). 7

Fig. S2. SEM image of Ag NPs prepared at 40 ^{o}C without HNT. 7

Fig. S3. TEM images of Au-Ag@HNTs with the adding Au:Ag atomic ratios of 0.5 (a), 1 (b), and 5 (c). (d) UV-Vis spectra of Au-Ag@HNTs with different Au:Ag atomic ratios. 8

Fig. S4. The content of Ag and Au(Pt) in Au(Pt)-Ag@HNT with different adding Au(Pt):Ag atomic ratios measured by Inductively Coupled Plasma Optical Emission Spectrometry (ICP-OES). 8

Fig. S5. TEM images of Pt-Ag@HNTs with the adding Pt:Ag atomic ratios of 0.5 (a),

and 1 (b). 9
Fig. S6. Powder X-ray diffraction (XRD) patterns of HNT, Ag@HNT, Au-Ag@HNT, and Pt-Ag@HNT..9
Fig. S7. The DAP formation rate constant *k of Au-Ag@HNT (a) and Pt-Ag@HNT (b) in four cycles.**...10
Fig. S8. Peroxidase-like catalytic performance of Au-Ag@HNT and Pt-Ag@HNT generated in different atomic ratios**.**...10
Fig. S9. Time dependent UV-vis absorption spectra for the catalytic reduction of 4-NP by $NaBH_4$ in the presence of (a) Ag@HNT (7.5 mg), (b) Au-Ag@HNT (4.5 mg) and (c) Pt-Ag@HNT (4.5 mg). 11
Fig. S10. TEM images of Au NPs (a), Pt NPs (b), Au@HNT (c), and Pt@HNT (d). 11

References. 12

1. Preparation of Au NPs and Au@HNT

10 mM $HAuCl_4$ aqueous solution (0.8 mL) was added to PVP aqueous solution (0.5 mg mL^{-1}, 40 mL) in 50 ^oC water bath. Then, AA aqueous solution (100 mM, 0.16 mL) was slowly dropped into the above solution under vigorously magnetic stirring. The color of the solution was changed from light yellow to red within several seconds, showing the formation of Au NPs. The reaction mixture was stirred for 10 min and centrifuged at 12000 rpm for 15 min.

For the synthesis of Au@HNT, 10 mM $HAuCl_4$ aqueous solution (0.8 mL) and 8 mg HNT were added to PVP aqueous solution (0.5 mg mL^{-1}, 40 mL) in 50 ^oC water bath. Then, AA aqueous solution (100 mM, 0.16 mL) was slowly dropped into the above solution under vigorously magnetic stirring. The color of the solution was changed from light yellow to purple within several seconds, indicating the formation of Au@HNT. The reaction mixture was stirred for 10 min and centrifuged at 9000 rpm for 15 min.

The as-prepared Au NPs and Au@HNT were washed twice with deionized water, and dried at 65 ^oC in oven overnight. TEM images of Au NPs and Au@HNT were shown in Fig. S10.

2. Preparation of Pt NPs and Pt@HNT

10 mM $H_2PtCl_6·6H_2O$ aqueous solution (2 mL) was added to deionized water (40 mL) at room temperature. Then, $NaBH_4$ aqueous solution (100 mM, 0.4 mL) was slowly dropped into the above solution under vigorously magnetic stirring. The color of the solution was changed from light yellow to brown within several seconds due to the formation of Pt NPs. The reaction mixture was stirred for 30 min and centrifuged at 12000 rpm for 15 min.

For the synthesis of Pt@HNT, 10 mM $H_2PtCl_6{\cdot}6H_2O$ aqueous solution (2 mL) and 8 mg HNT were added to PVP solution (0.5 mg mL^{-1}, 40 mL) at room temperature. Then, $NaBH_4$ aqueous solution (100 mM, 0.4 mL) was slowly dropped into the above solution under vigorously magnetic stirring. The color of the solution was changed from light yellow to dark brown within several seconds, indicating the formation of Pt@HNT. The reaction mixture was stirred for 30 min and centrifuged at 9000 rpm for 15 min.

The as-prepared Pt NPs and Pt@HNT were washed twice with deionized water and ethanol, respectively, and dried at 65 ^{o}C in oven overnight. TEM images of Pt NPs and Pt@HNT were shown in Fig. S10.

Table S1. The experimental parameters in the synthesis of bimetallic nanoparticles@HNTs. *

	Ag@HNT(mg)	$HAuCl_4$(μL)	Adding Au:Ag atomic ratio
Au-Ag@HNT	5.2	200	0.1
	2.6	500	0.5
	2.6	1000	1
	Ag@HNT(mg)	H_2PtCl_6(μL)	Adding Pt:Ag atomic ratio
Pt-Ag@HNT	5.2	200	0.1
	2.6	500	0.5
	2.6	1000	1

*Ag@HNT and various amount of 10 mM $HAuCl_4$(H_2PtCl_6) were dispersed in PVP solution (1 mg mL^{-1}, 100 mL) in 50 ^{o}C water bath to prepare Au(Pt)-Ag@HNT.

Table S2. The 4-NP reduction rate constant *k** in the presence of Ag@HNT prepared at 40 ^{o}C, 60 ^{o}C, and 80 ^{o}C before and after heating at 400 ^{o}C under nitrogen.

Preparation Temperature(^{o}C)	Thermal treatment Temperature(^{o}C)	k^*(s^{-1} mol^{-1})
40	RT	20.5
	400	351.0
60	RT	34.6
	400	191.2
80	RT	35.6
	400	63.7

Table S3. The comparison of DAP formation rate constant *k** (normalized based on the mole of active elements) in our catalytic systems and that reported in literatures. Au(Pt)-Ag@HNT was prepared with the adding atomic ratio Au(Pt):Ag of 0.1.

Sample	k^* (mol-DAP min^{-1} mol-M^{-1})	Data source
Au-Ag@HNT (0.1)	0.39	our work
Pt-Ag@HNT (0.1)	0.99	our work
AuAg NPs	0.12	He's work[1]
PtAg NPs	0.11	He's work[1]

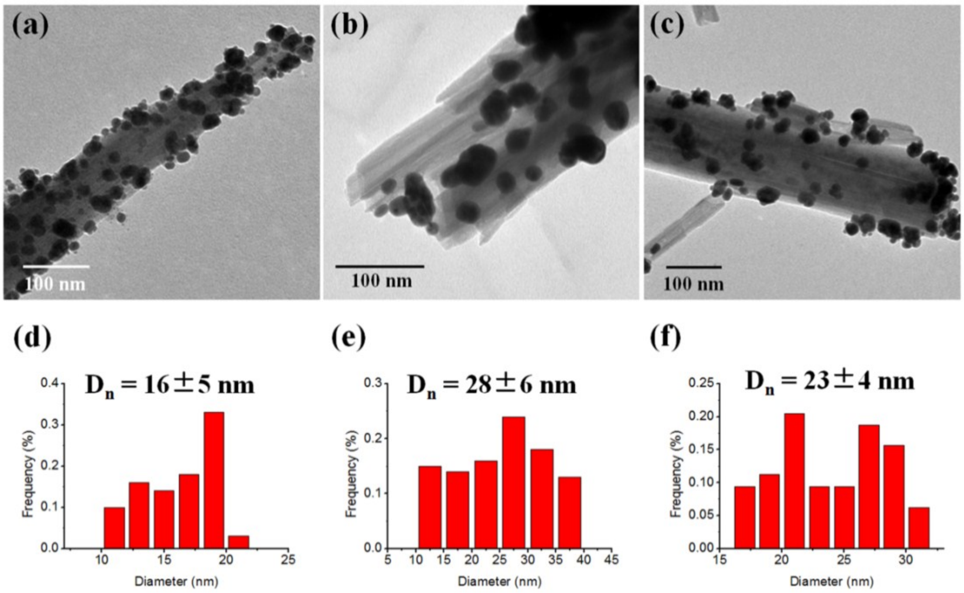

Fig. S1. (a, b, c) TEM images of Ag@HNTs prepared at 60 °C (a), 80 °C (b), and 100 °C (c). (d, e, f). Size distribution of Ag nanoparticles prepared at 60 °C (d), 80 °C (e), and 100 °C (f).

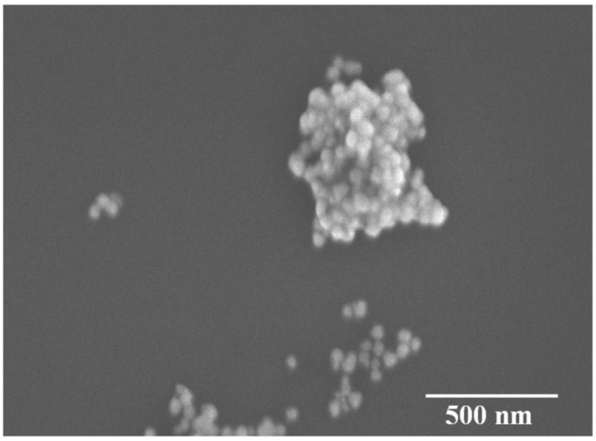

Fig. S2. SEM image of Ag NPs prepared at 40 °C without HNT.

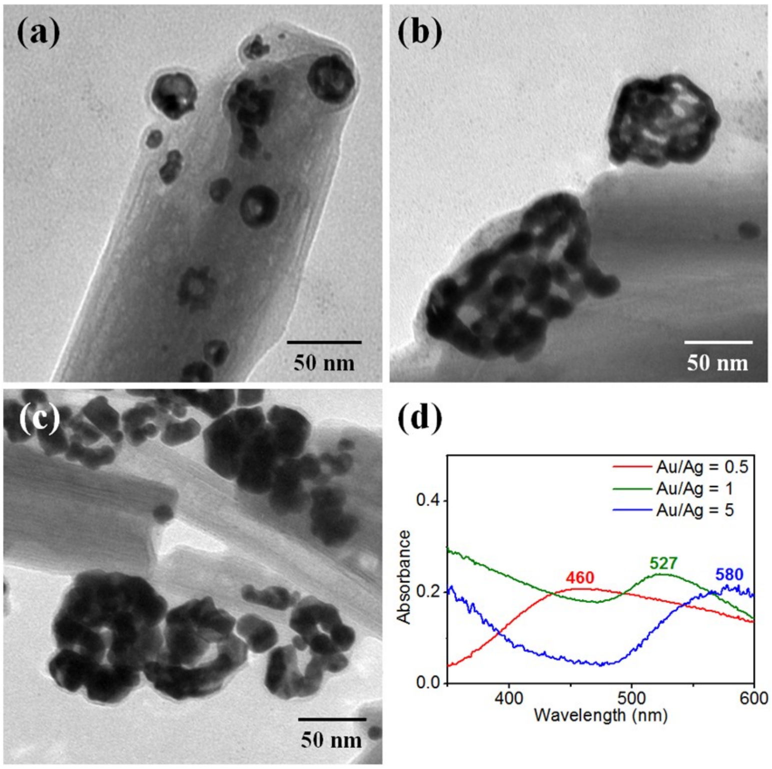

Fig. S3. TEM images of Au-Ag@HNTs with the adding Au:Ag atomic ratios of 0.5 (a), 1 (b), and 5 (c). (d) UV-Vis spectra of Au-Ag@HNTs with different Au:Ag atomic ratios.

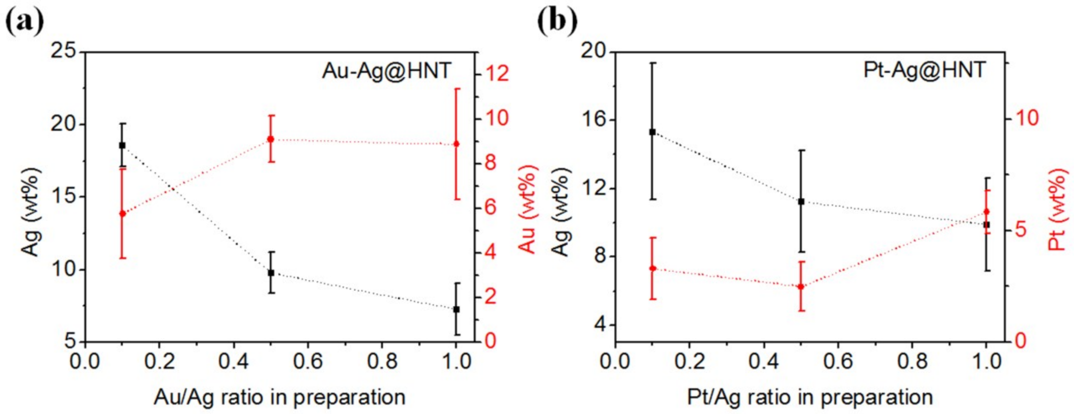

Fig. S4. The content of Ag and Au(Pt) in Au(Pt)-Ag@HNT with different adding Au(Pt):Ag atomic ratios measured by Inductively Coupled Plasma Optical Emission Spectrometry (ICP-OES).

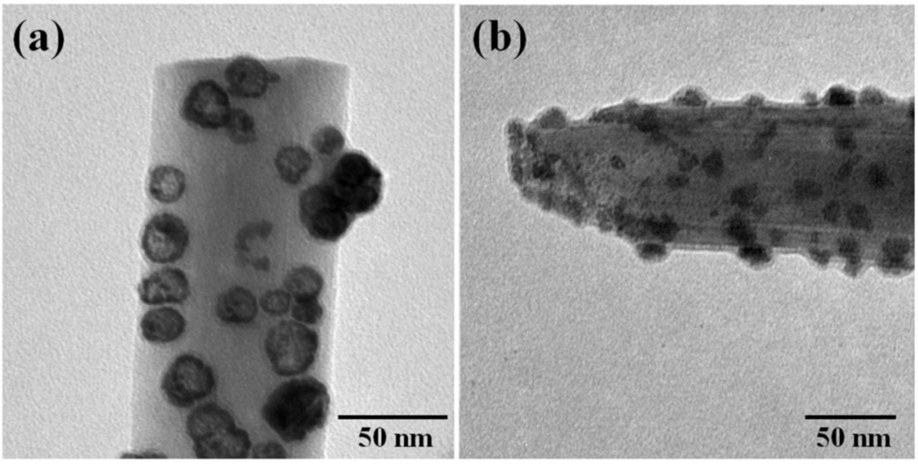

Fig. S5. TEM images of Pt-Ag@HNTs with the adding Pt:Ag atomic ratios of 0.5 (a), and 1 (b).

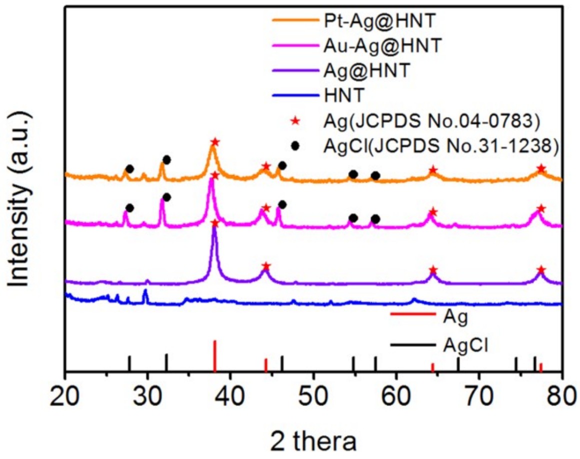

Fig. S6. Powder X-ray diffraction (XRD) patterns of HNT, Ag@HNT, Au-Ag@HNT, and Pt-Ag@HNT. Compared with standard patterns of Ag and AgCl. Au(Pt)-Ag@HNT was prepared with the adding atomic ratio Au(Pt):Ag of 0.1.

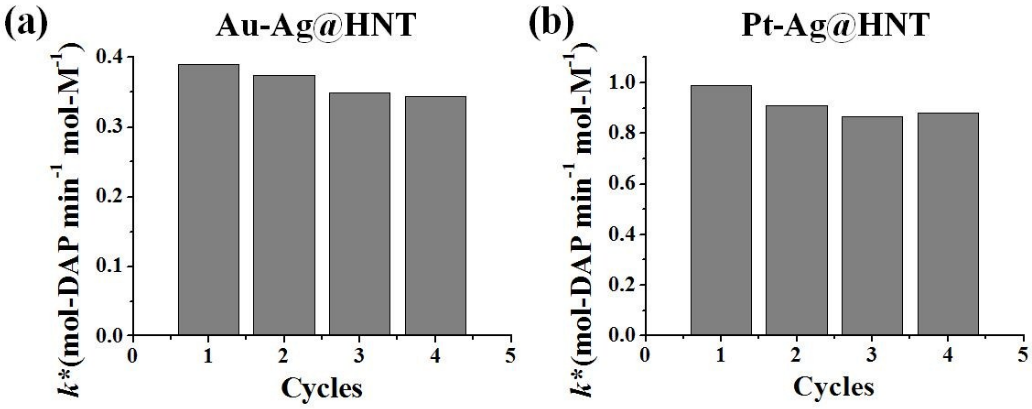

Fig. S7. The DAP formation rate constant *k** of Au-Ag@HNT (a) and Pt-Ag@HNT (b) in four cycles. Reaction conditions: H_2O_2 (0.3 M), OPD (0.3 mM), and catalysts (0.2 mg) at 40 ^oC. Au(Pt)-Ag@HNT was prepared with the adding atomic ratio Au(Pt):Ag of 0.1.

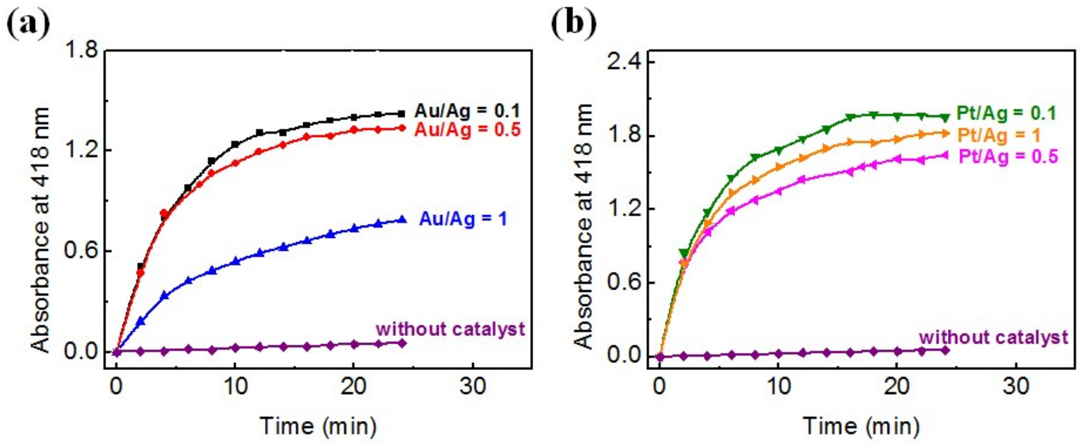

Fig. S8. Peroxidase-like catalytic performance of Au-Ag@HNT and Pt-Ag@HNT generated in different atomic ratios. Absorbance at 418 nm as a function of time was measured in the presence of Au-Ag@HNT (a) and Pt-Ag@HNT (b). The purple line marks the value of the control experiment. Reaction conditions: H_2O_2 (0.3 M), OPD (0.3 mM), and catalyst (0.2 mg) at 40 ^oC.

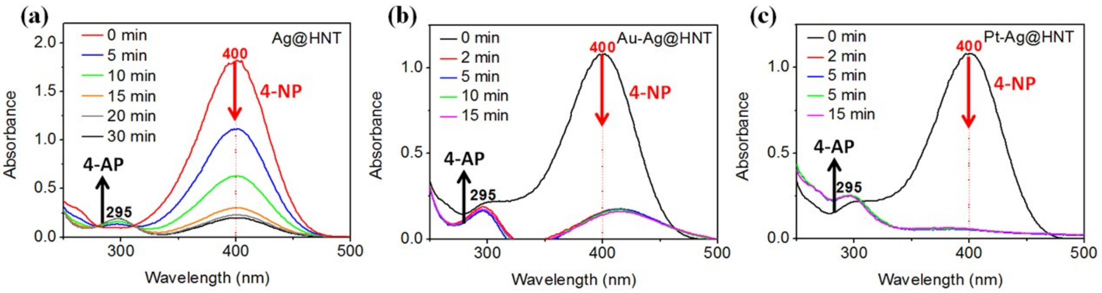

Fig. S9. Time dependent UV-vis absorption spectra for the catalytic reduction of 4-NP by $NaBH_4$ in the presence of (a) Ag@HNT (7.5 mg), (b) Au-Ag@HNT (4.5 mg) and (c) Pt-Ag@HNT (4.5 mg). Au(Pt)-Ag@HNT was prepared with the adding atomic ratio Au(Pt):Ag of 0.1.

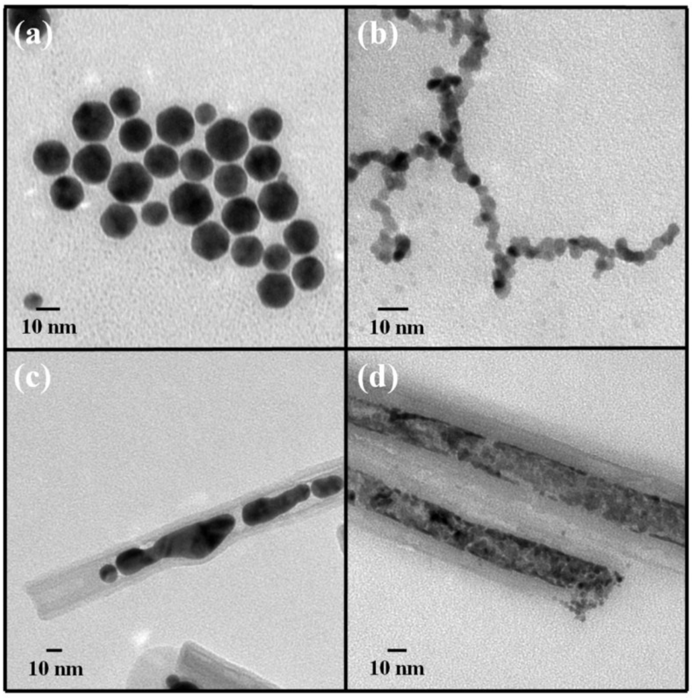

Fig. S10. TEM images of Au NPs (a), Pt NPs (b), Au@HNT (c), and Pt@HNT (d).

References

1 He, W.; Wu, X.; Liu, J.; Hu, X.; Zhang, K.; Hou, S.; Zhou, W.; Xie, S., Design of AgM Bimetallic Alloy Nanostructures (M = Au, Pd, Pt) with Tunable Morphology and Peroxidase-Like Activity. *Chem. Mater.* **2010,** *22* (9), 2988-2994.
